# Biphasic Regulation of Epithelial Antimicrobial Peptides During *Candida albicans* Vaginal Infection: Distinct Contributions of NLRP3/IL-1β and IL-17RA Pathways to β-Defensin-1 and -3 Expression

**DOI:** 10.3390/jof12030204

**Published:** 2026-03-11

**Authors:** Sofía Carla Angiolini, Emilse Rodriguez, Clarisa Manzone-Rodriguez, Paula Alejandra Icely, María Soledad Miró, Fernando Oscar Riera, Pablo Iribarren, Juan Pablo Caeiro, Claudia Elena Sotomayor

**Affiliations:** 1 Laboratory of Innate Immunity to Fungal Pathogens, Department of Clinical Biochemistry, Faculty of Chemical Sciences, National University of Córdoba, Córdoba 5000, Argentina; sangiolini@unc.edu.ar (S.C.A.); emirodriguez@unc.edu.ar (E.R.); paula.icely@mi.unc.edu.ar (P.A.I.); msmiro@unc.edu.ar (M.S.M.); 2 Center for Research in Clinical Biochemistry and Immunology, CIBICI-CONICET, National University of Córdoba, Córdoba 5000, Argentina; 3 Research Group of Immunology and Mycology, Córdoba 5000, Argentina; 4 Division of Infectious Diseases, Sanatorio Allende, Córdoba 5000, Argentina; 5 Division of Infectious Diseases, AltaMed Health Service Corporation, Los Angeles, CA 90040, USA

**Keywords:** *Candida albicans*, vulvovaginal candidiasis, antimicrobial peptide, β-defensin, NLRP3/IL-1β signaling, IL-17RA signaling

## Abstract

*Candida albicans* is the primary agent of acute vulvovaginal candidiasis (VVC) and its recurrent form (RVVC). Local innate immunity contributes to both defense and pathogenesis during vaginal *Candida* infection, where epithelial β-defensins (BD) constitute key components of the mucosal barrier. We previously reported that epithelial BD-1 expression is dynamically modulated during murine and human vaginitis, revealing strain-dependent and stimulus-specific regulation but leaving the host pathways involved unresolved. This study functionally defines the contribution of key immune pathways to epithelial antimicrobial peptide regulation. Using a murine model of VVC and the virulent *C. albicans* strain SC5314, we aimed to evaluate the immune signaling pathways governing the temporal regulation of epithelial BD-1 and BD-3 expression during vaginal infection. In wild-type mice, both defensins displayed a biphasic pattern: early induction followed by attenuation as infection progressed. Genetic loss-of-function approaches revealed that NLRP3/IL-1β signaling is required for early BD-1 induction, whereas IL-17RA signaling preferentially supports sustained BD-3 expression. Together, these findings establish a causal and temporal link between host immune signaling and epithelial defensin regulation and reveal a transient subversion of mucosal defenses by *C. albicans*. This work advances understanding of epithelial innate immunity, defining distinct temporal programs for BD-1 and BD-3 and identifying NLRP3/IL-1β and IL-17RA signaling as key pathways shaping mucosal defensin expression.

## 1. Introduction

*Candida albicans* is a common commensal fungus of the human mucosal microbiota and the main etiological agent of vulvovaginal candidiasis (VVC). This acute inflammatory disease affects approximately 75% of women at least once during their reproductive lifetime and represents one of the most common causes of vaginal infection, following bacterial vaginosis. Between 6% and 10% of these women experience a distressing condition characterized by recurrent episodes of VVC (RVVC), defined as four or more symptomatic episodes per year [1,2,3]. Estimates indicate that RVVC affects around 138 million women worldwide annually, underscoring its significant public health impact [4]. Despite the high prevalence of *Candida* vaginitis, many aspects of the host–fungal interaction remain poorly understood [5].

Antifungal immune responses in the vaginal mucosa are distinct from those observed in other *Candida* infections. In this particular tract, the fungus coexists under a tightly regulated surveillance mechanism involving resident cells and basal levels of immune mediators [6]. During asymptomatic vaginal colonization, epithelial cells (ECs) sense its presence through surface-expressed pattern recognition receptors (PRRs) that recognize *Candida* as a harmless commensal [5,7]. The *C. albicans* yeast-to-hypha transition marks the shift to pathogenicity and triggers virulence programs that drive tissue invasion [7,8,9], epithelial activation, antimicrobial peptide (AMP) expression, a strong interleukin-1 beta (IL-1β)-driven inflammatory response, and polymorphonuclear leukocyte (PMN) recruitment [10,11,12].

Among the cytokines involved in antifungal defense at mucosal sites, IL-1β and interleukin-17 (IL-17) play pivotal roles in orchestrating local immune responses [13,14]. IL-1β, produced by epithelial and innate immune cells upon fungal recognition, is a key mediator of inflammation and mucosal protection and is considered a hallmark of VVC. In parallel, IL-17 signaling has been implicated in antifungal immunity, in part by promoting epithelial activation and PMN recruitment. However, the contribution of IL-17 to VVC remains controversial, as several studies have reported low or undetectable levels of this cytokine in the vaginal environment, suggesting that its role may be limited or context-dependent [15,16].

AMPs are evolutionarily conserved effector molecules that display broad antimicrobial activity against pathogens. Among them, β-defensins (BDs) constitute one of the most important AMP family members [17,18]. These small (4–6 kDa) cationic peptides not only exert potent microbicidal effects but also act as immune modulators by recruiting PMNs, immature dendritic cells, and memory T cells to infection sites [18,19,20]. Human Beta Defensin 1 (hBD1) is constitutively expressed in the uninflamed mucosa of the female reproductive tract and is considered a major epithelial defense factor against invading microorganisms. In contrast, inducible defensins such as hBD3 are rapidly upregulated in response to inflammatory or infectious stimuli, contributing to the reinforcement of mucosal protection [21,22].

We recently demonstrated that *C. albicans* modulates epithelial BD-1 expression during vaginal infection. In a murine VVC model, BD1 plays a dynamic role during infection, while in patients with RVVC, BD1 expression was markedly reduced at both mRNA and protein levels. Mechanistically, we showed that *C. albicans*-secreted aspartyl proteinases and lipases contribute to this downregulation, identifying a fungal immune evasion strategy that may facilitate recurrent infection [23]. While these findings provided relevant descriptive and correlative data, the host immune pathways governing this regulation remained undefined. Here, using mice deficient in NLRP3/IL-1β (*Nlrp3^(−/−)^*) and IL-17RA (*Il17ra^(−/−)^*) signaling in a murine VVC model, we sought to define the specific contribution of these pathways to epithelial BD-1 and BD-3 expression in the vaginal mucosa.

## 2. Methods

### 2.1. Candida albicans

The *Candida albicans* SC5314 strain was used in all experiments. This strain is considered highly virulent based on its well-documented ability to express multiple virulence-associated traits, including elevated secreted aspartyl protease (SAP) and lipase activities, strong adherence to epithelial surfaces, and robust biofilm-forming capacity, as reported in previous studies [24]. These features are known to contribute to epithelial invasion and immune activation [16,25,26]. Yeast cells were cultured on Sabouraud Dextrose Agar (SDA; Britania, Los Patos, Buenos Aires, Argentina) at room temperature (RT).

For infection assays, the inoculum for the experiments was standardized by counting yeast cells in phosphate-buffered saline (PBS) using a Neubauer hemocytometer (Boeco, Hamburg, Germany). The number of viable yeasts was determined in triplicate via plating on SDA and incubating for 48 h at RT.

### 2.2. Mice

C57BL/6J wild-type (WT) mice were purchased from the Faculty of Veterinary Science, National University of La Plata, Argentina. *Nlrp3^(−/−)^* and *Il17ra^(−/−)^* mice were used to genetically define the contribution of inflammasome- and IL-17-dependent pathways to epithelial antimicrobial peptide regulation during vaginal infection.

Knockout mice with targeted disruption of the IL17-RA receptor (*Il17ra^(−/−)^*) on the C57BL/6 background were provided by Amgen Inc. (Thousand Oaks, CA, USA) (MTA#7939181). Knockout mice with targeted disruption of the NLRP3 receptor (nlrp3/Jtm1Bhk) on the C57BL/6 background were obtained from The Jackson Laboratory (Bar Harbor, ME, USA).

All animals were maintained under a standard light cycle (12 h light/dark) with free access to food and water. All experimental procedures were approved and conducted in accordance with the guidelines of the Animal Experimentation Ethics Committee of the Faculty of Chemical Sciences, National University of Córdoba (Approval number: RD-2022-2789-E-UNC-DEC#FCQ) and strictly followed the recommendations of the Guide to the Care and Use of Experimental Animals published by the Canadian Council on Animal Care (OLAW Assurance number A5802-01). Every effort was made to minimize animal suffering during the experiments.

### 2.3. In Vivo VVC Model

Female WT, *Il17ra^(−/−)^*, and *Nlrp3^(−/−)^* mice (8–10 weeks old) were treated subcutaneously with 0.2 mg of β-estradiol 17-valerate (Merck-Millipore, Darmstadt, Germany) dissolved in 100 μL of sesame oil (Merck-Millipore) on days −6 and −3, before vaginal infection on day 0. On days 2 and 4 post infection (pi), mice received additional estradiol injections to maintain the pseudoestrus state [27]. Estrogenized mice were then inoculated intravaginally with 20 μL of a *C. albicans* SC5314 suspension (infected group; 5 × 10^6^ viable yeasts/PBS), a dose selected during model standardization, or PBS (uninfected group) [26]. All infected WT mice were included in CFU analyses (*n* = 22), which served as the primary readout to confirm infection; sample size for each additional analysis depended on cervicovaginal lavage recovery and assay availability and is indicated in the corresponding figure legends.

For each experimental condition, WT mice were included as internal controls and processed in parallel with *Il17ra^(−/−)^* or *Nlrp3^(−/−)^* animals. WT and knockout mice were infected, sampled, and analyzed within the same experimental runs to ensure direct comparability. *Nlrp3^(−/−)^* and *Il17ra^(−/−)^* mice were included at *n* = 5 per group; the sample size for each additional parameter is indicated in the corresponding figure legends.

### 2.4. CVL Collection

Mice were anesthetized with isoflurane, and cervicovaginal lavages (CVLs) were collected by gently washing the vaginal lumen with 70 μL of sterile PBS [27,28]. This procedure allows recovery of vaginal luminal cells (including epithelial cells and recruited leukocytes), as well as soluble mediators present in the vaginal fluid, as previously described in murine models of vaginal candidiasis [29]. The collected CVLs were immediately processed for quantification of CFUs or PMNs. Another portion of each CVL was centrifuged for 10 min at 4 °C, and the resulting cell-free supernatant was stored at −80 °C for subsequent cytokine analysis.

### 2.5. Fungal Burden (CFU/mL)

CVL samples were serially diluted (1:10, 1:100) in sterile PBS, and 100 μL aliquots of each dilution were plated in duplicate onto Petri dishes containing SDA. Plates were incubated at RT for 48 h, and the number of yeast colonies was recorded as colony-forming units per milliliter (CFU/mL) [26,30]. For clarity of data presentation, WT control groups corresponding to each knockout experiment are shown separately in the figures, as they were processed in parallel under identical experimental conditions.

### 2.6. PMN Quantification

Cells from CVL samples were adjusted to a concentration of 5 × 10^4^ cells per 100 μL by means of resuspension in PBS, based on total cell counts determined using a Neubauer hemocytometer. Subsequently, samples were subjected to cytocentrifugation at 500 rpm for 5 min onto glass slides using a Shandon Elliot cytospin system (Shandon Scientific Co., Ltd., London, UK). The cells were then stained with May–Grünwald Giemsa (MGG; Biopak, Buenos Aires, Argentina) and examined under a NIKON ECLIPSE microscope (Nikon, Tokyo, Japan). PMNs were identified based on morphological criteria. For each sample, PMNs were manually counted in ten non-adjacent fields at a 40× objective. PMN counts were averaged across fields for each sample, and results were expressed as mean PMN counts per group ± standard error of the mean (SEM) [26].

### 2.7. Cytokine Quantification

Cytokine concentrations in CVL samples were quantified using commercially available IL-1β and IL-17A ELISA detection kits (R&D Systems, McKinley Place, Minneapolis, MN, USA; Mouse IL-1β, Cat. No. DY401-05; Mouse IL-17A, Cat. No. DY421-05), as previously described [26,31]. Absorbance was measured using a BIO-RAD microplate reader (Bio-Rad Laboratories, Hercules, CA, USA), and cytokine concentrations were interpolated from standard curves. Data were expressed as pg/mL.

### 2.8. Immunofluorescence for the Evaluation of mBD1 and mBD3 Expression

To detect BDs by means of immunofluorescence, 10 μL of CVL from each experimental group was placed on glass slides and air-dried at RT. Samples were fixed with pure methanol (Sintorgan, Villa Martelli, Argentina) for 5 min at RT and washed with PBS. Blocking was performed with 10% bovine serum albumin (BSA) and 0.3% Triton X-100 in PBS for 2 h at RT. Slides were then incubated overnight (ON) at 4 °C with a rabbit IgG primary antibody against mouse BD-1 (Santa Cruz Biotechnology, Dallas, TX, USA, Cat. No. sc-25573) or mouse BD-3 (Santa Cruz Biotechnology, Cat. No. sc-25573) (1:50). After three PBS washes, samples were incubated for 2 h at RT with a rabbit anti-IgG secondary antibody conjugated to Alexa Fluor 488 (Invitrogen, Carlsbad, CA, USA, Cat. No. A-11008) at a 1:500 dilution.

Slides were mounted with FluorSave™ Reagent (Merck Millipore). Images were acquired using a LEICA DMi8 inverted fluorescence microscope (Leica Microsystems, Wetzlar, Germany) equipped with a 20× objective. At least ten images were captured per cytospin preparation, each containing a minimum of ten cells per field. mBD1 and mBD3 expression was evaluated in the cytoplasm of vaginal ECs, identified in bright-field images based on their characteristic morphology. Selection masks were generated to individually delineate each cell and were superimposed onto the corresponding fluorescence images, allowing specific quantification of the cytoplasmic signal. This approach ensured accurate and cell-specific assessment of BD expression. Semi-quantitative analysis of mBD1 and mBD3 expression was performed using ImageJ software (National Institutes of Health, Bethesda, MD, USA; version 1.54p). Fluorescence intensity (FI) was expressed as the mean per-cell intensity normalized to the control group treated on day 2 of the kinetic study, according to the following equation:

$$\text{Normalized}\;\text{fluorescence}=\frac{\text{FI}\;\text{of}\;\text{infected}\;\text{mouse}\;\text{ECs}}{\text{Mean}\;\text{FI}\;\text{of}\;\text{day}\;2\;\text{treated}\;\text{control}\;\text{ECs}}$$

### 2.9. Statistical Analysis

Experiments were performed in triplicate unless otherwise indicated. Data were expressed as the mean ± standard error of the mean (SEM). Differences between groups were analyzed using parametric statistical tests, including Student’s *t*-test (for two-group comparisons) or one-way ANOVA, followed by appropriate post hoc tests (Dunnett’s, Bonferroni’s, or Welch’s), depending on the assumptions of normality and homogeneity of variance. Statistical significance was defined as *p* < 0.05 (*), *p* < 0.01 (**), *p* < 0.001 (***), and *p* < 0.0001 (****). All analyses were performed using GraphPad Prism 10 software (GraphPad, San Diego, CA, USA).

## 3. Results

### 3.1. Establishment of Infection and Local Inflammatory Profile During Experimental Vulvovaginal Candidiasis in C57BL/6 (WT) Mice

**Figure 1:**
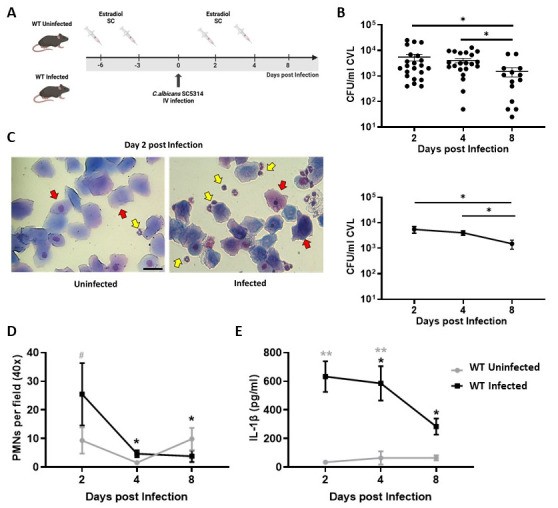
Establishment of Infection and Local Inflammatory Profile During Experimental Vaginal Candidiasis in C57BL/6 (WT) Mice. (**A**) Schematic representation of the murine VVC model in WT mice. (**B**) Individual (**top**) and mean (**bottom**) intravaginal fungal load, expressed as *C. albicans* CFU per mL of CVL, in Infected WT mice at days 2, 4, and 8 post infection. Values represent the mean ± SEM (*n* ≥ 14). Data were analyzed using the Mann–Whitney test at each specific time point. * p < 0.05. (**C**) Representative images of the cell populations present in CVL cytospin from Uninfected or Infected mice at day 2 post infection, stained with May–Grünwald Giemsa (×400 magnification). Red arrows indicate the ECs, and yellow arrows indicate the PMNs. (**D**) PMN counts from CVL cytospin preparations of Uninfected (gray line) and Infected (black line) WT mice at days 2, 4, and 8 post infection, stained with May–Grunwald Giemsa (×40 magnification). Values represent the mean ± SEM (*n* ≥ 10). (**E**) IL-1β levels in CVL samples from Uninfected (gray line) and Infected (black line) mice at days 2, 4, and 8 post infection, determined by means of ELISA. Values represent the mean ± SEM (*n* ≥ 6). (**D**,**E**) Data were analyzed using two-way ANOVA. Black asterisks indicate significant differences within the Infected group relative to day 2. Gray asterisks indicate significant differences between the Uninfected and Infected groups at the same time point. * *p* < 0.05. ** *p* < 0.01. ^#^ *p* = 0.06 (Uninfected and Infected, day 2). WT: wild-type; SC: subcutaneous; IV: intravaginal; CFU: Colony-Forming Units; CVL: Cervicovaginal lavage; PMNs: Polymorphonuclear neutrophils; ECs: Epithelial cells. SEM = standard error of the mean; ANOVA = analysis of variance.

To characterize the modulation and kinetic expression of mBD1 and mBD3 during VVC, we first explored the key local parameters associated with the vaginal mucosal response to *C. albicans* SC5314 infection in C57BL/6 (WT) mice. Estrogen-treated WT animals were intravaginally infected with 5 × 10^6^ viable yeast on day 0 (Infected group), while a control group received PBS (uninfected group) (Figure 1A). CVLs were collected on days 2, 4, and 8 post infection (pi) for subsequent analysis. First, to assess the progression of *C. albicans* infection, local fungal burden was quantified by CFUs on each day of the study. WT-infected mice displayed robust colonization that persisted throughout the evaluated period, with similar values on days 2 and 4, followed by a significant decrease in fungal burden by day 8 (Figure 1B). MGG-stained slides of cells present in CVL from both groups of animals revealed abundant ECs, consistent with shedding of superficial epithelial layers and estrogen-induced epithelial turnover (Figure 1C). In this model, we observed a peak recruitment of PMNs in WT-infected mice at day 2, followed by a marked decrease at days 4 and 8, showing significant differences compared with the uninfected group (Figure 1D).

In line with the aim of the present study, we evaluated the intravaginal concentrations of IL-1β and IL-17A. We observed that the highest levels of IL-1β in CVL from infected mice were detected at days 2 and 4 (Figure 1E) with a significant decrease by the end of the study (day 8). These increases were significant compared with the uninfected WT group at all evaluated time points (Figure 1E). Regarding IL-17A, our findings showed low local production of this cytokine in both infected and uninfected WT animals, with IL-17A concentrations remaining below 15 pg/mL, which corresponds to the detection limit of the assay.

### 3.2. C. albicans SC5314 Infection Differentially Modulates mBD1 Expression in the Vaginal Tract

**Figure 2:**
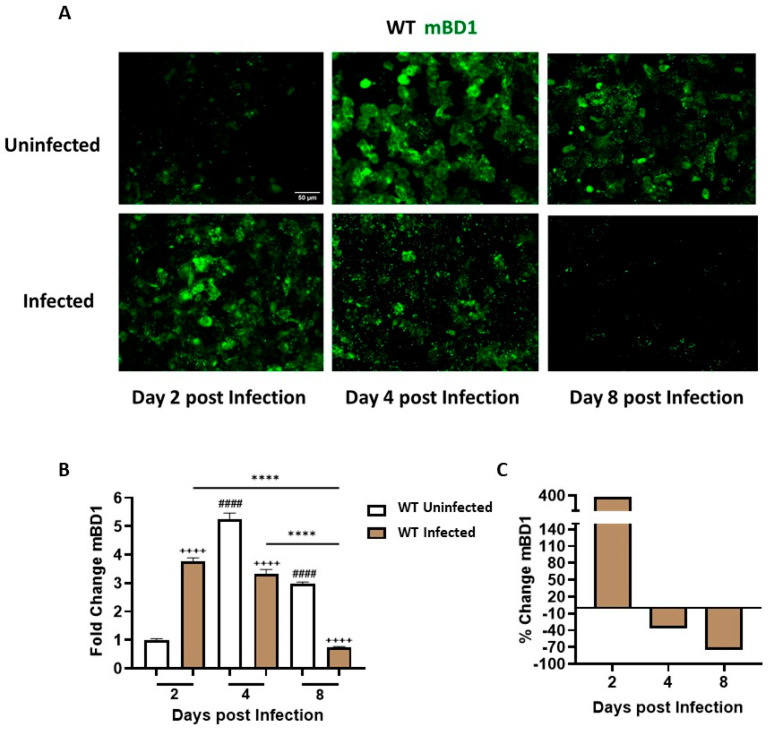
Differential expression of Beta Defensin-1 (mBD1) in ECs from cervicovaginal lavage cells during VVC. (**A**) Representative images of CVL cytospin preparations from Uninfected and Infected WT mice at days 2, 4, and 8 post infection. mBD1 protein expression was detected by means of indirect immunofluorescence (IF) using a rabbit anti-mouse BD-1 primary antibody and an Alexa Fluor 488-conjugated anti-rabbit IgG secondary antibody. Images were captured with a LEICA DMi8 inverted fluorescence microscope (×200 magnification). mBD1 expression appears in green. (**B**) Semi-quantification of mBD1 fluorescence intensity, normalized to the Uninfected group at day 2 post infection and expressed as fold change. Analysis was performed using ImageJ software (*n* ≥ 4 mice per time point). Values represent the mean ± SEM. Data were analyzed using one-way ANOVA. ^++++^ *p* < 0.0001 (Infected vs. Uninfected, days 2, 4, and 8). **** *p* < 0.0001 (Infected, days 2 and 4 vs. day 8). ^####^ *p* < 0.0001 (Uninfected, days 4 and 8 vs. day 2). (**C**) Fluorescence intensity in the Infected group expressed as a percentage change in mBD1 relative to the Uninfected group at days 2, 4, and 8 post infection. CVL: Cervicovaginal lavage. WT: wild-type. SEM = standard error of the mean; ANOVA = analysis of variance.

mBD1 protein expression was assessed in ECs recovered from CVL of infected and uninfected WT mice using a specific anti-mBD1 antibody and IF assay (Figure 2A). We observed distinct kinetic profiles in each experimental group associated with exposure to treatments (Figure 2B). At day 2 pi, infected animals exhibited a significant increase in mBD1 expression compared with the uninfected group, showing an approximately 380% rise, indicating an early and strong response to *C. albicans* presence (Figure 2C). At day 4 pi, mBD1 expression levels in infected animals were similar to those at day 2 but showed a significant decrease compared with the uninfected group, highlighting the marked response of the vaginal tract to estrogenic treatment and the ability of the fungus to inhibit peptide expression. Analysis of percentage variation indicated a 37% reduction in the infected group (Figure 2C). By the end of the study (day 8), while estrogen treatment continued to stimulate mBD1 expression, infection progression resulted in a 75% reduction in peptide levels. These results illustrate the mucosal tract’s ability to upregulate peptide expression early during infection (day 2), as well as the capacity of *C. albicans* to inhibit mBD1 as the infection advances.

These results deepen our earlier observations by demonstrating that mBD1 modulation reflects a coordinated and temporally regulated epithelial response during vaginal infection.

### 3.3. C. albicans SC5314 Infection Differentially Modulates mBD3 Expression in the Vaginal Tract

**Figure 3:**
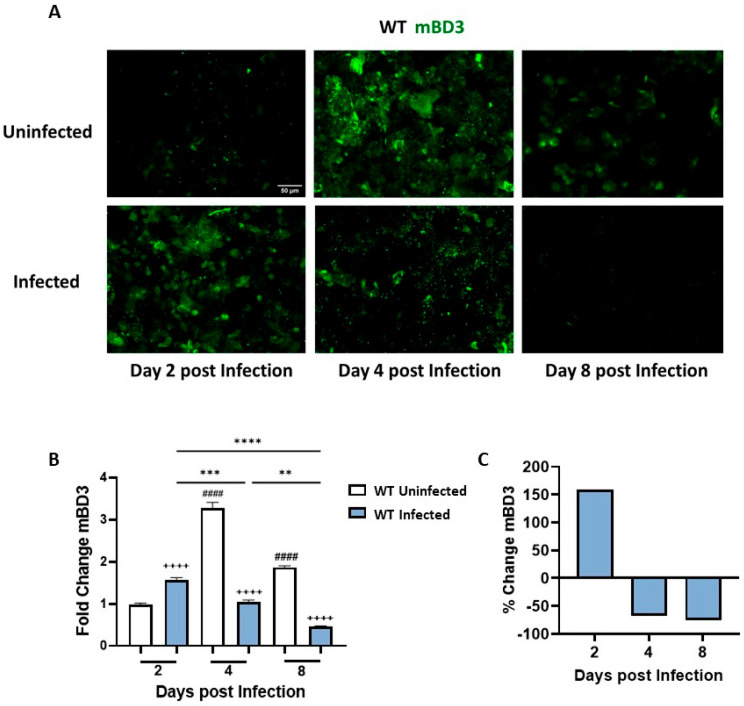
Differential expression of Beta Defensin-3 (mBD3) in ECs from cervicovaginal lavage cells during VVC. (**A**) Representative images of CVL preparations from Uninfected and Infected WT mice at days 2, 4, and 8 post infection. mBD3 protein expression in ECs was detected by means of indirect immunofluorescence (IF) using a rabbit anti-mouse BD-3 primary antibody and an Alexa Fluor 488-conjugated anti-rabbit IgG secondary antibody. Images were obtained with a LEICA DMi8 inverted fluorescence microscope (×200 magnification). mBD3 expression appears in green. (**B**) Semi-quantification of mBD3 fluorescence intensity, normalized to the Uninfected group at day 2 post infection and expressed as fold change. Analysis was performed with ImageJ software (*n* ≥ 4 mice per time point). Values represent the mean ± SEM. Data were analyzed using one-way ANOVA. ^++++^ *p* < 0.0001 (Infected vs. Uninfected days 2, 4, and 8). ** *p* < 0.01 (Infected day 4 vs. day 8). *** *p* < 0.001 (Infected day 2 vs. day 4). **** *p* < 0.0001 (Infected day 2 vs. day 8). ^####^ *p* < 0.0001 (Uninfected days 4 and 8 vs. day 2). (**C**) Fluorescence intensity of the Infected group expressed as a percentage change in mBD3 relative to the Uninfected group at days 2, 4, and 8 post infection. CVL: cervicovaginal lavage. WT: wild-type. SEM = standard error of the mean; ANOVA = analysis of variance.

mBD3 expression was studied in ECs recovered from CVL of infected and uninfected WT mice using a specific anti-mBD3 antibody and IF assay (Figure 3A). At the early time point of the kinetic study (day 2), mBD3 exhibited a peak of expression associated with the presence of *C. albicans*, with a significant difference compared with the uninfected group (Figure 3B). The percentage variation analysis indicated an increase of 159% in mBD3 expression relative to the control group (Figure 3C). At day 4 pi, mBD3 expression levels in infected mice showed a significant decrease compared with day 2 pi and with the uninfected group. The percentage variation between groups at day 4 revealed a 68% reduction in peptide expression in infected animals compared with uninfected controls. Interestingly, estrogen treatment also stimulates mBD3 expression in this tract, reaching its highest levels at this time point (Figure 3B). By day 8 of the study, estrogen treatment continued to stimulate mBD3 expression in the uninfected group, whereas infection progression led to an approximately 75% reduction in mBD3 expression (Figure 3C).

Together, these findings indicate that mBD3 exhibits a biphasic expression pattern during *C. albicans* infection, with early induction followed by sustained downregulation, similar to that observed for mBD1.

### 3.4. NLRP3 Inflammasome Pathway Activation Drives mBD1 but Not mBD3 Induction During C. albicans Vaginal Infection

**Figure 4:**
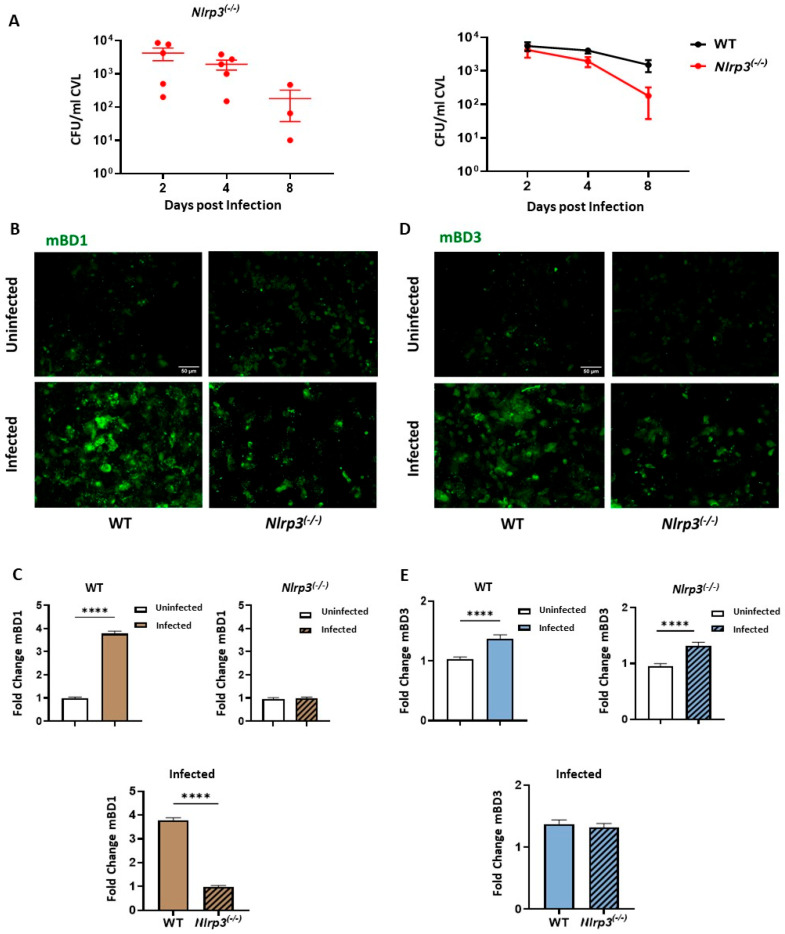
Regulation of Beta Defensin-1 (mBD1) and Beta Defensin-3 (mBD3) in *Nlrp3^(−/−)^* mice during VVC. (**A**) Individual intravaginal fungal load counts, expressed as *C. albicans* CFUs/mL of CVL, in Infected *Nlrp3^(−/−)^* mice at days 2, 4, and 8 post infection (**left**). Comparative analysis of CFU/mL between WT (black line) and *Nlrp3^(−/−)^* (red line) mice throughout infection kinetics (**right**). Values represent the mean ± SEM (*n* ≥ 3). Data were analyzed using the Mann–Whitney test at each specific time point and two-way ANOVA for comparisons between WT and *Nlrp3^(−/−)^* animals. (**B**,**D**) Representative images of CVL cytospin preparations from WT and *Nlrp3^(−/−)^* mice, Uninfected and Infected at day 2 post-infection. ECs were stained by means of IF to detect mBD1 (**B**) or mBD3 (**D**) expression. Images were captured using a LEICA DMi8 inverted fluorescence microscope (×200 magnification). mBD1 and mBD3 expression is shown in green (top panels). (**C**,**E**) The lower panels show semi-quantification of mBD1 (**C**) and mBD3 (**E**) fluorescence intensity, normalized to the Uninfected group at day 2 post infection and expressed as fold change in WT and *Nlrp3^(−/−)^* mice, comparing infected and uninfected groups, using ImageJ software (*n* = 5 mice). Values represent the mean ± SEM. Data were analyzed using Student’s *t*-test. **** *p* < 0.0001. WT: wild-type; CFU: Colony-Forming Units; CVL: Cervicovaginal lavage; ECs: Epithelial cells. SEM = standard error of the mean; ANOVA = analysis of variance.

To genetically define the contribution of NLRP3/IL-1β-dependent signaling to epithelial AMP regulation during VVC, we developed the experimental model described above in NLRP3-deficient mice (*Nlrp3^(−/−)^*) and compared their responses with those obtained from WT animals. For this purpose, estrogen-treated *Nlrp3^(−/−)^* animals were iv infected with 5 × 10^6^ viable *C. albicans* SC5314 yeasts on day 0 (Infected *Nlrp3^(−/−)^* group), while a control group received PBS inoculation (uninfected *Nlrp3^(−/−)^* group). CVLs were collected on days 2, 4, and 8 pi to assess the progression of fungal infection based on CFU counts. *Nlrp3^(−/−)^* mice exhibited robust vaginal colonization by *C. albicans*, which persisted throughout the evaluation period (Figure 4A, left panel). Fungal burdens in infected *Nlrp3^(−/−)^* mice were comparable to those observed in WT animals at days 2 and 4, followed by a significant reduction at day 8 (Figure 4A, right panel).

Based on these findings, day 2 pi was selected to investigate the contribution of NLRP3/IL-1β signaling to mBD1 and mBD3 regulation. At this time point, both peptides exhibited peak expression in infected WT mice, while fungal burden remained comparable between WT and *Nlrp3^(−/−)^* groups, allowing comparison independent of colonization differences. In addition, estrogen-mediated stimulation of defensin expression becomes more prominent at later time points, allowing infection-driven regulation to be examined independently of hormonal effects. Accordingly, CVL from both genotypes was collected on day 2 of the experimental schema. Figure 4B shows a representative image of mBD1 expression in ECs recovered from infected and uninfected mice, and Figure 4C displays the corresponding fold change in peptide expression. As observed, *C. albicans* infection induced a significant increase in mBD1 expression in WT animals compared with uninfected animals. In contrast, *Nlrp3^(−/−)^* mice showed comparable mBD1 expression levels in infected and uninfected groups. Moreover, when comparing infected animals of both genotypes, *Nlrp3^(−/−)^* mice exhibited significantly lower mBD1 expression than WT mice.

For mBD3, similar to what was observed in infected WT animals (Figure 4D,E), *Nlrp3^(−/−)^* mice showed a significant increase in mBD3 levels in response to *C. albicans* infection compared with the uninfected *Nlrp3^(−/−)^* group. Interestingly, comparison between infected groups of both genotypes revealed that mBD3 levels in the *Nlrp3^(−/−)^* group were comparable to those observed in WT mice (Figure 4E).

Collectively, these findings indicated that NLRP3/IL-1β signaling is required for optimal mBD1 induction during early infection, whereas mBD3 regulation occurs independently of this pathway.

### 3.5. IL-17RA Signaling Differentially Regulates mBD1 and mBD3 Expression During C. albicans Vaginal Infection

**Figure 5:**
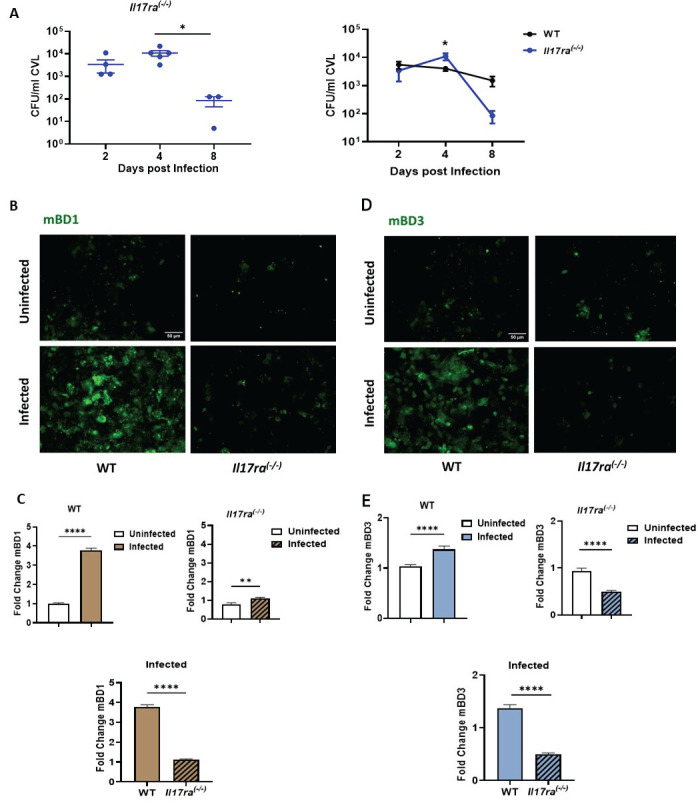
Regulation of Beta Defensin-1 (mBD1) and Beta Defensin-3 (mBD3) in *Il17ra^(−/−)^* mice during VVC. (**A**) Individual intravaginal fungal load counts, expressed as *C. albicans* CFU per mL CVL, in Infected *Il17ra^(−/−)^* mice at days 2, 4, and 8 post infection (**left**). Comparative analysis of CFU/mL between WT (black line) and *Il17ra^(−/−)^* (blue line) mice during infection kinetics (**right**). Values represent the mean ± SEM (*n* ≥ 3). Data were analyzed using the Mann–Whitney test at each specific time point and two-way ANOVA for comparisons between WT and *Il17ra^(−/−)^* animals. * *p* < 0.05. (**B**,**D**) Representative images of CVL cytospin preparations from WT and *Il17ra^(−/−)^* mice, Uninfected and Infected at day 2 post infection. ECs were stained by means of IF to detect mBD1 (**B**) or mBD3 (**D**) expression. Images were captured with a LEICA DMi8 inverted fluorescence microscope (×200 magnification). mBD1 and mBD3 expression is shown in green (top panels). (**C**,**E**) The lower panels show the semi-quantification of mBD1 (**C**) and mBD3 (**E**) fluorescence intensity, normalized to the Uninfected group at day 2 post infection and expressed as fold change in WT and *Il17ra^(−/−)^* mice, comparing infected and uninfected groups, using ImageJ software (*n* = 4 mice). Values represent the mean ± SEM. Data were analyzed using Student’s *t*-test. ** *p* < 0.01, **** *p* < 0.0001. WT: wild-type; CFU: Colony-Forming Units; CVL: Cervicovaginal lavage; ECs: Epithelial cells. SEM = standard error of the mean; ANOVA = analysis of variance.

While IL-17 plays a well-established role in intestinal candidiasis [14], its contribution to the immunopathogenesis of VVC remains unclear [15,16]. To evaluate the role of IL-17RA signaling in vaginal infection and BD regulation, the VVC model described above was established in IL-17RA-deficient mice (*Il17ra^(−/−)^*). Estrogen-treated *Il17ra^(−/−)^* animals were iv infected with 5 × 10^6^ viable *C. albicans* SC5314 yeasts on day 0 (Infected *Il17ra^(−/−)^* group), while a control group received PBS inoculation (uninfected *Il17ra^(−/−)^* group). The progression of infection was evaluated based on CFUs in the CVL collected on days 2, 4, and 8 pi (Figure 5A, left panel). No significant differences in fungal burden were observed between infected *Il17ra^(−/−)^* mice at days 2 and 4 pi, but at day 8, these animals showed a decrease in fungal burden compared to previous days. When compared with infected WT mice, no significant differences in CFUs were observed between both strains at day 2. A transient increase in vaginal fungal burden was observed in *Il17ra^(−/−)^* mice at day 4 pi; however, this difference was no longer evident by day 8. (Figure 5A, left panel). At the end of the study (day 8), infected *Il17ra^(−/−)^* mice showed a reduction in fungal burden compared to the WT group.

Since fungal burden at day 2 remained similar between WT and *Il17ra^(−/−)^* mice, and both mBD1 and mBD3 expression at this time point are independent of estrogenic stimulation, we selected this day to evaluate the contribution of IL-17 signaling to BD regulation in the vaginal tract after *C. albicans* infection (Figure 5B,D). As previously demonstrated in WT mice, fungal infection induced a significant increase in mBD1 expression compared to uninfected controls. In *Il17ra^(−/−)^* mice, *C. albicans* infection also resulted in a significant increase in mBD1 expression relative to uninfected *Il17ra^(−/−)^* animals. However, comparison between infected groups of both genotypes revealed that mBD1 levels in *Il17ra^(−/−)^* mice were significantly lower than those observed in WT animals.

For mBD3, meanwhile, *C. albicans* infection induced an increase in peptide expression at day 2 pi compared with the uninfected WT control. *Il17ra^(−/−)^* mice failed to upregulate mBD3 expression in response to *C. albicans* infection, showing significantly lower levels compared with infected WT mice (Figure 5D,E). Finally, when comparing both genotypes, infected *Il17ra^(−/−)^* mice exhibited significantly lower mBD3 levels than infected WT animals.

Collectively, these findings indicate that IL-17RA signaling contributes to mBD3 induction during *C. albicans* vaginal infection, whereas mBD1 expression is only partially dependent on this pathway.

## 4. Discussion

The onset of clinical symptoms in patients with VVC is characterized by the presence of *Candida* in the vaginal exudate, a local inflammatory reaction driven by PMN recruitment into the lumen, and high concentrations of proinflammatory cytokines, with IL-1β being considered pathognomonic of this infection [7,32]. The VVC model in C57BL/6 mice infected with *C. albicans* SC5314 used in this study reproduces these key infection parameters. The fungus was isolated from the vaginal cavity throughout the course of infection. A high fungal burden was present at days 2 and 4 pi, accompanied by a strong recruitment of PMNs into the vaginal lumen (day 2) and elevated IL-1β levels. Despite their abundance, PMNs fail to control *C. albicans* growth, and their recruitment does not correlate with fungal clearance [33,34]. Rather than promoting fungal elimination, PMNs recruited to the vaginal lumen contribute to tissue damage and amplify local inflammation [12]. Evidence from murine and human studies indicates that neutrophil depletion does not modify fungal burden and that components of the vaginal environment, such as heparan sulfate, can neutralize PMNs’ fungicidal activity [7,35]. Thus, although neutrophils are classically regarded as key effector cells, their antimicrobial function appears limited in the vaginal milieu [3,5], underscoring the relevance of exploring additional microbicidal mechanisms, such as the contribution of epithelial-derived AMPs.

Studies in the human female genital tract have demonstrated that hBDs are key components of the mucosal defense system, acting as broad-spectrum AMPs that contribute to the control of viral, bacterial, parasitic, and fungal pathogens [36,37]. In addition to their antimicrobial role, their expression is modulated by hormones. Estrogen-dependent upregulation of BD-1 through ERα and ERβ signaling pathways has also been demonstrated [38,39]. In line with this concept, our results showed a peak in the expression of both the constitutive mBD1 and the inducible mBD3 at days 4 and 8 in estrogen-treated but uninfected WT mice. These results provide novel data on the interplay between hormonal signaling and epithelial antimicrobial responses in the murine vaginal mucosa.

Both mBD1 and mBD3 exhibit direct candidacidal activity, although with different efficacies. In vitro studies have shown that mBD3 is the more potent fungicidal peptide, retaining activity under physiological salt conditions and acting against both yeast and hyphal forms, whereas mBD1 is weaker and more salt-sensitive [40,41]. Beyond their antimicrobial properties, these peptides exert distinct immunoregulatory functions. mBD1 contributes to mucosal homeostasis by maintaining epithelial integrity and controlling commensal balance, thereby limiting excessive inflammation [36,42]. In contrast, mBD3 displays a broader immunomodulatory profile, enhancing cytokine and chemokine production and promoting the recruitment of PMNs, dendritic cells, and T cells. Moreover, its interaction with PRR signaling pathways suggests a dual role in amplifying antifungal responses while modulating immune activity under certain inflammatory or chronic conditions [43].

An important finding from our time-course studies of vaginal infection reveals a dynamic epithelial response, as the vaginal epithelium senses *C. albicans* and mounts a robust upregulation of both mBD1 and mBD3 by day 2 pi, reflecting an early barrier-driven attempt to contain fungal growth. However, by days 4 and 8, the fungus markedly suppresses the expression of both the constitutive mBD1 and the inducible mBD3. This biphasic pattern reflects our previous clinical observations in women with VVC, where ECs from acute cases showed high hBD1/hBD3 expression, but patients with RVVC exhibited strong transcriptional and protein downregulation. Consistently, in vitro experiments using SAP and LIP inhibitors demonstrated that these fungal virulence factors contribute to this regulatory switch [23]. Notably, although infection is commonly associated with AMP induction, defensin levels at later stages were lower than those observed in estrogen-treated uninfected controls. This finding suggests that sustained hormonal stimulation alone is sufficient to maintain BD expression. In contrast, during infection, fungal virulence mechanisms progressively counteract epithelial defensin production, reinforcing the concept of active immune evasion. In this context, it is important to note that BD expression in this study was assessed at the protein level in cells recovered from the vaginal lumen, a sampling approach used in our study in both acute and recurrent VVC patients due to its minimally invasive nature and ease of collection, while reliably reflecting the local mucosal immune microenvironment. This strategy provides biologically relevant information on the availability of AMPs at the mucosal interface, where host–fungus interactions occur. Previous studies have shown that BD transcript levels do not necessarily correlate with protein abundance in VVC, supporting the relevance of protein-based analyses [23]. Moreover, the evaluation of defensin expression in vaginal lavage cells allows the identification of responding cellular subsets, an aspect that cannot be captured by bulk transcriptomic approaches.

Similar to our results, an inhibitory pattern of BDs expression has been reported in other genital tract infections. In women infected with *Chlamydia trachomatis* or *Neisseria gonorrhoeae*, levels of hBD1, hBD2, and hBD3 in CVL were significantly lower than in uninfected controls [44,45]. In a larger cohort of women with bacterial vaginosis, BD expression was also reduced, particularly for hBD2 [44]. This inhibitory effect on BD production is not restricted to the genital tract. In gastric and intestinal epithelia, *Helicobacter pylori*, *Vibrio cholerae*, and enterotoxigenic *Escherichia coli* have been shown to downregulate hBD1 through virulence factors such as CagA or bacterial toxins [46,47,48]. Given the critical antimicrobial and immunomodulatory functions of epithelial BDs, their suppression can represent an efficient and conserved mechanism of immune evasion at mucosal surfaces. Our findings in patients and experimental models clearly demonstrate that *C. albicans* exploits this strategy to establish and persist in vaginal tracts, weakening local defenses and promoting conditions that favor the permanence of infection.

Having established the epithelial response pattern in WT animals infected with *C. albicans* SC5314, we next wanted to functionally define the contribution of key immune pathways to epithelial AMP regulation. mBD1 and mBD3 expression were assessed in mice deficient in NLRP3 and IL-17RA using the VVC model. The canonical NLRP3/IL-1β pathway has been well documented in animal models [8,49] and in patients with NLRP3 polymorphisms [50]. The canonical IL-1β pathway, activated through fungal pathogen-associated molecular patterns (PAMPs) and NLRP3 inflammasome signaling, plays a central role in cytokine secretion [51]. We previously reported that recombinant human IL-1β induces hBD1 expression in human ECs from the female genital tract, supporting a role for IL-1β in BD-1 regulation [23]. Pietrella et al. [52] reported that components of the NLRP3 inflammasome pathway were overexpressed in vaginal samples from patients with VVC, demonstrating that inflammasome activation occurs during *Candida* vaginitis. Similarly, NLRP3 induction was observed in vaginal tissue following murine *C. albicans* infection [51,53]. Nevertheless, our results in infected *Nlrp3^(−/−)^* mice revealed that the absence of NLRP3 did not impair the control of vaginal fungal burden, as these mice showed colonization levels comparable to WT animals at early time points and even lower CFU counts at the end of the study. Thus, NLRP3 signaling is dispensable for the control of vaginal fungal burden under the experimental conditions tested. Consistently, inhibition of NLRP3 with glyburide in infected WT animals mimicked the results observed in *Nlrp3^(−/−)^* mice [51], further supporting that NLRP3 signaling is dispensable for fungal clearance in this mucosal site.

To evaluate the contribution of the NLRP3 pathway to the functional regulation of BDs, we next examined the expression of mBD1 and mBD3. Day 2 pi was selected for these analyses, as both peptides showed a clear increase in response to *C. albicans* at this time point. Notably, this represents an early phase of infection when estrogen-driven stimulation is not detected and before the fungus exerts its inhibitory effects on defensin expression. Although NLRP3 was nonessential for fungal control, its absence markedly affected the epithelial response. The significant reduction in mBD1 expression in infected *Nlrp3^(−/−)^* mice compared with WT animals indicates that mBD1 induction is strongly dependent on NLRP3/IL-1β signaling. This result supports a model in which infection-driven inflammasome activation and IL-1β release act as upstream cues for the transcriptional upregulation of constitutive epithelial defensin. In contrast, mBD3 expression was preserved in *Nlrp3^(−/−)^* mice, suggesting that its induction follows an inflammasome-independent pathway. Given that mBD3 is an inducible peptide typically associated with inflammatory stimuli, these findings highlight distinct regulatory networks governing mBD1 and mBD3 expression during the early response to *C. albicans* challenge. Altogether, our data reveal that while NLRP3/IL-1β signaling is essential for optimal mBD1 expression, alternative mechanisms are sufficient to trigger mBD3 upregulation in the vaginal tract. In agreement, previous evidence positions IL-1β as a major regulator of BD-1. Pahl et al. [54] reported in a *Candida* esophagitis infection model that blockade of IL-1β receptor-dependent signaling strongly reduces the induction of hBDs. They also describe a signaling loop between IL-1β and hBDs regulation. Several in vitro results have confirmed this report [55,56]. We likewise demonstrated that recombinant hIL-1β markedly upregulates hBD1 expression in hECs from the female genital tract, identifying IL-1β as a key regulator of this peptide [23]. Loss of NLRP3 selectively impaired early BD-1 induction, providing genetic evidence that inflammasome-dependent signaling governs the initial epithelial antimicrobial response suggested by our prior in vitro observations. Together, these data support IL-1β–mediated signaling as a central link between fungal sensing and epithelial antimicrobial responses in the vaginal tract.

Consistent with previous reports, the absence of IL-17RA signaling did not impair the control of *C. albicans* in the vaginal mucosa. *Il17ra^(−/−)^* mice showed fungal kinetics similar to WT animals early in infection and a faster decline at later stages. These observations align with studies demonstrating that IL-17–mediated responses, while essential for antifungal protection at oral and intestinal sites [13,14], play a limited or dispensable role in VVC [15,16]. The tolerogenic nature of the vaginal mucosa, together with estrogen-mediated modulation, could explain the limited contribution of IL-17 to fungal clearance [57,58]. Despite this, IL-17RA signaling contributes to the regulation of BD. Our results identify mBD3 as the defensin most strongly associated with IL-17RA-dependent signaling during vaginal *C. albicans* infection. The marked reduction in mBD3 expression in *Il17ra^(−/−^*^)^ mice, compared with the partial effect observed for mBD1, suggests that IL-17–mediated pathways play a dominant and selective role in driving BD-3 transcription. Notably, this regulatory role was observed even though IL-17 remained undetectable in CVL samples from infected mice, suggesting that cytokine production is stringently controlled within the vaginal mucosa and may occur at levels below detection in vaginal fluids. Such a scenario could reflect transient or spatially restricted IL-17 expression, limited recruitment of IL-17-producing cells to the vaginal tissue, or modulation by local immune regulatory mechanisms [26,59,60]. Collectively, these observations emphasize the finely tuned and compartmentalized nature of IL-17 responses in the mucosal vaginal tract.

In our model, the absence of IL-17RA signaling strongly impaired mBD3 expression, supporting a central role for IL-17-mediated pathways in its induction during vaginal *C. albicans* infection. Similar observations have been reported in other mucosal sites where IL-17 enhances BD expression. Verma et al. [61] demonstrated that Th17 cytokines can activate epithelial and immune cells to release alarmins and AMPs, including BDs. Likewise, IL-17 stimulation increased mBD3 expression in murine colonic epithelial cells [62], and in nasal tissue, IL-17A promoted mBD3 upregulation in response to *Staphylococcus aureus* colonization [63]. Also, it was reported that IL-17 potently induces BD through epithelial activation of NF-κB, MAPK, and C/EBP pathways, which are essential for the transcription of AMP genes such as *Defb2* and *Defb3* [64,65]. The partial induction of mBD1 in *Il17ra^(−/−^*^)^ mice suggests that additional regulatory pathways, including the NLRP3/IL-1β axis, contribute to its expression, as also demonstrated in this work. Complementary to this, prior studies have demonstrated that IFN-α, activated during acute viral challenge, rapidly upregulates hBD1 in human immune cells [66], indicating a direct link between IFN-I signaling and BD-1 transcriptional induction. Interestingly, other authors and our group have reported that IFN-I is relevant in mucosal defense in *Candida* vaginitis [25,26,67]. Overall, the data presented herein provide a framework for future mechanistic studies of defensin regulation during VVC.

In summary, this study reveals a dynamic interplay between host epithelial defenses and *C. albicans* during vaginal infection, defining distinct regulatory programs for constitutive and inducible BDs throughout the course of VVC. Our findings demonstrate that cytokine-driven pathways differentially control mBD1 and mBD3 in the vaginal mucosa, uncovering nonredundant mechanisms of epithelial antimicrobial regulation. Specifically, we propose a biphasic model in which NLRP3/IL-1β signaling predominates early mBD1 induction, whereas IL-17RA-dependent pathways are preferentially associated with mBD3 regulation during vaginal infection. By integrating genetic deficiency models with temporal analyses, this work provides strong evidence supporting a regulatory role for NLRP3/IL-1β and IL-17RA pathways in epithelial AMP expression during vaginal candidiasis and provides new insight into the temporal coordination of mucosal innate immunity, laying a conceptual foundation for the development of targeted or combinatorial therapeutic strategies against VVC.

## Data Availability

The original contributions presented in this study are included in the article. Further inquiries can be directed to the corresponding author.
